# Increased Risk for Substance Use-Related Problems in Autism Spectrum Disorders: A Population-Based Cohort Study

**DOI:** 10.1007/s10803-016-2914-2

**Published:** 2016-10-12

**Authors:** Agnieszka Butwicka, Niklas Långström, Henrik Larsson, Sebastian Lundström, Eva Serlachius, Catarina Almqvist, Louise Frisén, Paul Lichtenstein

**Affiliations:** 1 Department of Medical Epidemiology and Biostatistics, MEB, Karolinska Institutet, Box 281, 171 77 Stockholm, Sweden; 2 Department of Child Psychiatry, Medical University of Warsaw, Warsaw, Poland; 3 Department of Neuroscience, Uppsala University, Uppsala, Sweden; 4 Department of Medical Sciences, Örebro University, Örebro, Sweden; 5 Centre for Ethics, Law and Mental Health (CELAM), University of Gothenburg, Mölndal, Sweden; 6 Gillberg Neuropsychiatry Centre, University of Gothenburg, Gothenburg, Sweden; 7 Department of Clinical Neuroscience, Centre for Psychiatric Research, Karolinska Institutet, Stockholm, Sweden; 8 Child and Adolescent Psychiatry, Stockholm County Council, Stockholm, Sweden; 9 Lung and Allergy Unit, Astrid Lindgren Children’s Hospital, Stockholm, Sweden

**Keywords:** Autism spectrum disorder, Addiction, ADHD, Intellectual disability, ICD

## Abstract

Despite limited and ambiguous empirical data, substance use-related problems have been assumed to be rare among patients with autism spectrum disorders (ASD). Using Swedish population-based registers we identified 26,986 individuals diagnosed with ASD during 1973–2009, and their 96,557 non-ASD relatives. ASD, without diagnosed comorbidity of attention deficit hyperactivity disorder (ADHD) or intellectual disability, was related to a doubled risk of substance use-related problems. The risk of substance use-related problems was the highest among individuals with ASD and ADHD. Further, risks of substance use-related problems were increased among full siblings of ASD probands, half-siblings and parents. We conclude that ASD is a risk factor for substance use-related problems. The elevated risks among relatives of probands with ASD suggest shared familial (genetic and/or shared environmental) liability.

**Electronic supplementary material:**

The online version of this article (doi:10.1007/s10803-016-2914-2) contains supplementary material, which is available to authorized users.

## Introduction

Substance use-related problems have traditionally been considered rare in autism spectrum disorders (ASD), since the core features appeared to reduce the risk of using psychoactive substance. (Ramos et al. 2013; Santosh and Mijovic 2006) Yet, substance use-related problems have been observed among 19–30 % patients with ASD, at least in clinical settings (Hofvander et al. 2009; Sizoo et al. 2010). It has been suggested that the high rates of substance use-related problems may be attributed to comorbidity between ASD and attention deficit hyperactivity disorder (ADHD) (Palmqvist et al. 2014). Indeed, both ADHD and intellectual disability frequently co-occur with ASD (Hofvander et al. 2009; Buck et al. 2014) and are linked to substance use-related problems (Carroll Chapman and Wu 2012; Lee et al. 2011; Chang et al. 2014). Since psychiatric disorder comorbidity is more likely to be noted in highly selected clinical populations, the setting might considerably influence rates of concurrent, documented substance use-related problems (Hofvander et al. 2009; Jensen and Steinhausen 2014; Buck et al. 2014). One epidemiological (Abdallah et al. 2011) study found a similar risk of an alcohol abuse register-based diagnosis among 414 ASD individuals from the Danish Historic Birth Cohort compared to non-ASD controls. In contrast, recent data from two large, population-based twin cohorts suggested that autistic-like traits do increase the risk of substance use disorder (Lundstrom et al. 2011; De Alwis et al. 2014), implying that similar associations can be present in less selected samples of individuals with ASD.

We aimed to investigate the risk of substance use-related problems in ASD. We also tested if any association between ASD and substance use-related problems could be related to comorbidity with ADHD or intellectual disability (ID). To elucidate if shared familial factors underlie both ASD and substance use-related problems, we examined the pattern of substance use-related problems also among unaffected relatives of individuals with ASD.

## Methods

### Registers

We linked Swedish longitudinal, population-based registers: the National Patient Register, which contains all inpatient medical care (1973-) and outpatient, non-GP, specialist care (2001-), the Clinical Database for Child and Adolescent Psychiatry in the Stockholm County (Pastill) (Lundh et al. 2013), the Habilitation Register (Idring et al. 2012), the Swedish Prescribed Drug Register (2005-) (Wettermark et al. 2007), the Cause of Death Register (National Board of Health and Welfare 2009), the National Crime Register (National Council for Crime Prevention 2013), the Swedish Register of Education (Statistics Sweden 2011a), the National Censuses from 1960 to 1990 (Statistics Sweden 1992), the Integrated Database for Labor and Market Research (Statistics Sweden 2011b), the Total Population Register and the Multi-Generation Register (Ekbom 2011). Unique personal identification numbers, assigned to each Swedish resident, enabled data linkage across registers. National Swedish administrative medical registers contain systematically and longitudinally collected information due to mandatory reporting. Excellent diagnostic validity has been reported for many disorders; consequently, these registers have previously been used in many epidemiological investigations.

### Subjects

We identified 26,986 probands with an autism spectrum disorders (ASD) among all individuals born in Sweden between January 1, 1973 and December 31, 2009. ASD diagnoses from the National Patient, Pastill, or Habilitation Registers were defined according to WHO’s International Classification of Disease (ICD) (ICD-9 code 299; ICD-10 code F84). A prior validation study found that 96 % of register-based ASD diagnoses were consistent with ASD when medical journals were scrutinized (Idring et al. 2012).

### Relatives

We used linkage through the Multi-Generation Register to identify substance use-related problems among unaffected (without an ASD diagnosis) full siblings (N = 30,456), half-siblings (N = 15,946), and parents (N = 50,155) of probands with ASD.

### Outcome

Substance use-related problems were defined as one or more of: substance use disorder, any conviction for a substance-related crime, substance-related death (EMCDDA 2009), and alcohol-related somatic disease as defined in ICD (Table S1).

### Covariates

#### Co-morbidity

Analyses were stratified on probands’ psychiatric comorbidity with ADHD (ICD-9 code 314; ICD-10 codes F90.0, F90.1, F90.8 and F90.9) and/or ID (ICD-8 codes 310 to 315; ICD-9 codes 317–319; ICD-10 code F70-F93 and F79). Diagnostic data were extracted from the National Patient, Pastill, and Habilitation Registers. Pharmacotherapy (dispensed prescriptions) with stimulant (ATC codes N06BA01, N06BA02 or N06BA04) or non-stimulant (ATC code N06BA09) ADHD medications from the Swedish Prescribed Drug Register was also used to identify ADHD (Skoglund et al. 2014).

#### Socio-Demographic Covariates

Data on income and education were extracted from the Education Register, the LISA database and/or Censuses from 1970, 1975, 1980, 1985 and 1990. As an indicator of family economic status, we used disposable family income within the first 15 years of life, presented as population income percentile for the respective time period. The highest level of education obtained by either parent was used and the Migration Register provided data on parental country of birth. Missing data were not replaced but categorized as “unknown”.

### Statistical Analyses

#### Association Between Autism Spectrum Disorder and Substance Use-Related Problems

Similar to previous studies (Butwicka et al. 2014; Sullivan et al. 2012; Kyaga et al. 2011; Larsson et al. 2013) we used a matched cohort design to estimate the risk of substance use-related problems in two study population: probands with ASD and their relatives. Probands with ASD were matched on sex, birth year and county of birth to general population controls drawn from the Total Population Register. The number of controls for each ASD proband was restricted to 50 individuals randomly selected from the data set with matched individuals. Odds ratios (ORs) for each ASD proband were estimated from conditional logistic regression models stratified on matched sets to account for the matching by sex, birth year and county of birth. In analysis on relatives, full sibling, half-sibling and parents of probands with ASD were compared to matched relatives of non-ASD individuals, to full sibling controls, half-sibling controls, and parent controls, respectively. Multivariate analyses were adjusted for family income, parental education and country of origin. generalized estimating equations (GEEs) was used to correct for familial clustering of data.

When only familial confounding is considered, results from this method will be comparable to those from ordinary within-sibling analyses. In addition, we could adjust for non-familial confounding by using simple matching to population control (birth cohort effects, diagnostic patters different for gender and counties) with equal time at risk between the compared groups (bias due to left truncation or right censoring). (Lundstrom et al. 2014).

All statistical analyses were conducted with SAS software (version 9.3; Cary, NC, USA).

#### Sensitivity Analyses

First, we investigated the risk of substance-related problems among ASD probands in comparison to their population controls, separately for patients diagnosed with ASD on ICD-10 criteria and those diagnosed with ICD-8 or ICD-9. Second, to test whether there was any secular trend, we compared risks among ASD individuals born 1990–2009 with those born 1973–1989. Third, we investigated the effect of the timing of ADHD and/or ID diagnoses on risk of substance-related problems. Hence, the risk of substance-related problems was estimated separately for ASD probands who received all final neuropsychiatric diagnoses before substance use disorder was diagnosed. Analyses were undertaken separately for ASD probands without neuropsychiatric comorbidity, and with ADHD and/or ID.

The study was approved by the research ethics committee at Karolinska Institutet, Stockholm, Sweden Protocol nr 2009/5:10. No individual consent was needed since data were strictly register-based.

## Results

We identified 26,986 probands with an ASD diagnosis and compared them with 1,349,300 non-ASD individuals matched on sex, birth year and county of birth. The median age at the time of first ASD diagnosis was 13.8 years [interquartile range (IQR) = 8.8–18.4]. While 3.4 % (N = 913) of ASD patients had a preexisting substance use disorder diagnosis when diagnosed with ASD only 0.8 % (N = 10,789) of controls had a substance use disorder diagnosis when included in the study (p < 0.001). Descriptive variables differed slightly between groups with the most marked differences for parental age, education and family income (Table S2).

### Autism Spectrum Disorders and Risk of Substance Use-Related Problems

Probands had a substantially increased risk of any substance-related problem (OR 3.3; 95 % CI 3.1–3.6), such as substance use disorder (OR 5.2; 95 % CI 4.9–5.6), somatic disease linked to alcohol misuse (OR 5.9; 95 % CI 2.7–13.0), substance-related crime (OR 1.4; 95 % CI 1.2–1.5) and death (OR 3.0; 95 % CI 1.3–6.9). Within the substance use disorder category, the highest risk was found for drug use disorder (OR 8.5; 95 % CI 7.7–9.3), followed by tobacco (OR 6.4; 95 % CI 3.8–10.5) and alcohol use disorder (OR 4.0, 95 % CI 3.7–4.4). Adjustment for parental age, region of birth, education and family income did not change the results (Table 1). All risk estimates were elevated among ASD probands diagnosed with ICD-10 criteria, whereas probands diagnosed with earlier ICD versions, appeared less likely to develop substance-related problem compared to non-ASD individuals (OR 0.4; 95 % CI 0.2–0.6) (Table S3).

**Table 1:** Rates and odds ratios (with 95 % confidence interval) for substance use-related problems in autism spectrum disorder (ASD) probands and matched non-ASD population controls

Substance-related problem	Probands	Unexposed individuals	Univariate analysis	Multivariate analysis^a^
	Patients with ASD *N* = 26,986 *n* (%)	Non-ASD individuals *N* = 1,349,300 *n* (%)	Crude OR (95 % CI)	Adjusted OR (95 % CI)
Any problem	1079 (4.0)	17,643 (1.3)	3.3 (3.1–3.6)***	2.6 (2.4–2.7)***
Substance use disorder	980 (3.6)	10,228 (0.8)	5.2 (4.9–5.6)***	3.9 (3.6–4.2)***
Alcohol	574 (2.1)	7519 (0.6)	4.0 (3.7–4.4)***	3.1 (2.8–3.4)***
Drugs	579 (2.1)	3638 (0.3)	8.5 (7.7–9.3)***	5.6 (5.1–6.2)***
Tobacco	17 (0.1)	134 (0.0)	6.4 (3.8–10.5)***	4.6 (2.8–7.8)***
Crime	259 (1.0)	9687 (0.7)	1.4 (1.2–1.5)***	1.1 (1.0-1.2)
Somatic disease	7 (0.0)	59 (0.0)	5.9 (2.7–13.0)***	4.3 (1.9–9.7)***
Death	6 (0.0)	99 (0.0)	3.0 (1.3–6.9)**	2.0 (0.9–4.6)

***p value <0.001

^a^Adjustment for parental education, family income and substance use disorder prior to ASD diagnosis

**Table 2:** Odds ratios (with 95 % confidence interval) for substance use-related problems in ASD probands compared to matched non-ASD controls

Substance-related problem	Bivariate analysis				Multivariate analysis^a^			
	Comorbidity				Comorbidity			
	None	ADHD	ID	ADHD + ID	None	ADHD	ID	ADHD + ID
	Crude OR (95 % CI)	Crude OR (95 % CI)	Crude OR (95 % CI)	Crude OR (95 % CI)	Adjusted OR (95 % CI)	Adjusted OR (95 % CI)	Adjusted OR (95 % CI)	Adjusted OR (95 % CI)
Any problem	2.6 (2.4–2.9)***	8.3 (7.4–9.2)***	1.1 (0.9–1.3)	4.6 (3.7–5.8)***	2.2 (2.0-2.4)***	5.0 (4.5–5.6)***	1.0 (0.8–1.2)	3.3 (2.7–4.1)***
Substance use disorder	4.0 (3.6–4.5)***	12.4 (11.0-13.8)***	1.8 (1.4–2.2)***	7.4 (5.9–9.4)***	3.2 (2.9–3.6)***	7.2 (6.4–8.1)***	1.6 (1.3-2.0)***	5.3 (4.2–6.7)***
Alcohol	3.3 (2.9–3.8)***	8.2 (7.1–9.5)***	1.6 (1.2–2.1)***	6.1 (4.6–8.1)***	2.7 (2.4–3.1)***	5.0 (4.3–5.6)***	1.5 (1.2–1.9)**	4.6 (3.5–6.1)***
Drugs	6.0 (5.2-7.0)***	23.6 (20.3–27.3)***	2.5 (1.8–3.4)***	10.7 (7.9–14.6)***	4.4 (3.7–5.1)***	11.7 (10.0-13.7)***	2.2 (1.6-3.0)***	7.0 (5.1–9.6)***
Tobacco	2.4 (0.7–7.6)	15.8 (7.7–32.3)***	5.8 (1.7–19.1)***	3.8 (0.5–29.4)	2.0 (0.6–6.3)	7.4 (3.4–16.1)***	5.4 (1.6–17.7)**	2.8 (0.4–22.1)
Crime	1.0 (0.8–1.2)	4.0 (3.3–4.8)***	0.2 (0.1–0.4)***	1.4 (0.9–2.3)	0.9 (0.7–1.1)	2.6 (2.2–3.1)***	0.2 (0.1–0.3)***	1.1 (0.7–1.7)
Death	3.2 (1.0-10.3)	5.3 (1.2–22.6)*	–^b^	7.1 (0.9–58.1)	2.5 (0.8–8.2)	–^b^	–^b^	–^b^
Somatic disease	3.6 (0.9–15.0)	2.9 (0.4–22.1)	10.0 (2.2–45.6)**	25.0 (4.6 − 136.5)***	2.9 (0.7–12.3)	–^b^	–^b^	–^b^

Results stratified by comorbidity

*ADHD* attention deficit hyperactivity disorder, ID intellectual disability

*p value <0.05;**p value <0.01; ***p value <0.001

^a^Adjustment for parental education, family income and substance use disorder prior to ASD diagnosis

^b^OR and 95 % CI were not calculable due to no observations

**Table 3:** Rates and odds ratios (with 95 % confidence interval) for substance use-related problems in relatives of probands with autism spectrum disorders (ASD) compared to relatives of matched non-ASD controls

Relative	Substance-related problem	Rate *n* (%)	Total	Comorbidity in probands			
				None	ADHD	ID	ADHD + ID
			Crude OR (95 % CI)	Crude OR (95 % CI)	Crude OR (95 % CI)	Crude OR (95 % CI)	Crude OR (95 % CI)
Full siblings *N* = 30,456	Any problem	1191 (3.9)	1.5 (1.4–1.6)***	1.3 (1.2–1.5)***	1.8 (1.5-2.0)***	1.3 (1.1–1.5)**	1.9 (1.6–2.4)***
	Substance use disorder	831 (2.7)	1.6 (1.5–1.7)***	1.4 (1.2–1.6)***	2.0 (1.8–2.3)***	1.3 (1.1–1.6)***	2.1 (1.7–2.7)***
	Alcohol	605 (2.0)	1.5 (1.4–1.6)***	1.3 (1.1–1.5)***	1.9 (1.6–2.2)***	1.3 (1.0-1.5)*	2.1 (1.6–2.7)***
	Drugs	327 (1.1)	1.9 (1.7–2.1)***	1.7 (1.5–2.1)***	2.6 (2.1–3.2)***	1.4 (1.1–1.9)*	2.4 (1.6–3.4)***
	Tobacco	13 (0.0)	2.1 (1.1-4.0)*	2.0 (0.9–4.6)	2.0 (0.6–6.4)	3.1 (0.8–12.9)	–^a^
	Crime	530 (1.7)	1.3 (1.2–1.4)***	1.2 (1.0-1.4)*	1.5 (1.3–1.8)***	1.3 (1.0-1.6)*	1.6 (1.1–2.2)**
	Death	10 (0.0)	3.0 (1.3–6.7)**	1.9 (0.6–6.2)	7.4 (2.5–21.3)***	1.9 (0.4–7.7)	6.3 (0.8–51.4)
	Somatic disease	4 (0.0)	1.5 (0.6–4.2)	1.3 (0.3–5.4)	2.0 (0.3–15.2)	2.3 (0.3–17.2)	–^a^
Half siblings *N* = 15,946	Any problem	1264 (7.9)	1.2 (1.1–1.3)***	1.2 (1.1–1.3)**	1.3 (1.2–1.5)***	1.1 (0.9–1.2)	1.3 (1.1–1.6)**
	Substance use disorder	848 (5.3)	1.3 (1.2–1.4)***	1.2 (1.1–1.4)**	1.4 (1.2–1.6)***	1.2 (1.0-1.4)	1.5 (1.2–1.9)***
	Alcohol	589 (3.7)	1.2 (1.1–1.4)***	1.2 (1.0-1.4)*	1.3 (1.1–1.5)**	1.3 (1.0-1.6)*	1.2 (0.9–1.6)
	Drugs	401 (2.5)	1.4 (1.2–1.5)***	1.2 (1.0-1.4)*	1.5 (1.3–1.9)***	1.2 (0.9–1.5)	2.0 (1.5–2.7)***
	Tobacco	6 (0.0)	0.8 (0.3–1.7)	0.3 (0.0-1.9)	1.4 (0.4–4.6)	–^a^	2.2 (0.5–9.4)
	Crime	706 (4.4)	1.2 (1.1–1.3)***	1.2 (1.0-1.4)**	1.3 (1.1–1.5)***	1.0 (0.8–1.2)	1.1 (0.8–1.4)
	Death	12 (0.1)	1.4 (0.7–2.6)	1.5 (0.6–3.6)	1.7 (0.6–4.7)	–^a^	3.0 (0.9–9.4)
	Somatic disease	5 (0.0)	2.3 (0.9–5.9)	1.9 (0.4–8.3)	4.2 (0.9–19.2)	–^a^	3.8 (0.5–31.5)
Parents *N* = 50,155	Any problem	5720 (11.4)	1.5 (1.4–1.5)***	1.3 (1.2–1.3)***	2.0 (1.9–2.1)***	1.2 (1.1–1.3)***	1.7 (1.5–1.8)***
	Substance use disorder	3110 (6.2)	1.7 (1.6–1.8)***	1.4 (1.3–1.5)***	2.3 (2.2–2.5)***	1.4 (1.3–1.6)***	2.1 (1.9–2.4)***
	Alcohol	2401 (4.8)	1.7 (1.6–1.7)***	1.4 (1.3–1.5)***	2.2 (2.0-2.4)***	1.4 (1.3–1.6)***	2.2 (1.9–2.5)***
	Drugs	1187 (2.4)	1.9 (1.8-2.0)***	1.5 (1.3–1.6)***	2.9 (2.6–3.2)***	1.5 (1.3–1.7)***	2.2 (1.8–2.7)***
	Tobacco	137 (0.3)	1.5 (1.3–1.8)***	1.6 (1.2-2.0)***	2.0 (1.5–2.7)***	0.6 (0.3-1.0)	2.4 (1.4–4.1)**
	Crime	3855 (7.7)	1.3 (1.3–1.4)***	1.1 (1.1–1.2)***	1.9 (1.8-2.0)***	1.1 (1.0-1.2)	1.5 (1.3–1.6)***
	Death	161 (0.3)	1.8 (1.5–2.1)***	1.5 (1.2–1.9)**	2.7 (2.1–3.5)***	1.2 (0.8–1.8)	2.2 (1.2-4.0)**
	Somatic diseases	201 (0.4)	1.5 (1.3–1.8)***	1.3 (1.0-1.6)*	2.0 (1.5–2.6)***	1.7 (1.2–2.2)***	1.3 (0.8–2.3)

Results stratified by comorbidity in probands

*ADHD* attention deficit hyperactivity disorder, ID intellectual disability

*p value <0.05;**p value <0.01; ***p value <0.001

^a^OR and 95 % CI were not calculable due to no observations

Subsequently, we stratified analyses by ASD comorbidity with ADHD and/or ID (Table 2). Although ASD probands without such comorbidity also had increased risk of substance use-related problems (OR 2.6; 95 % CI 2.4–2.9), comorbid ADHD (OR 8.3; 95 % CI 7.4–9.2) or ADHD with ID (OR 4.6; 95 % CI 3.7–5.8) entailed a substantially higher risk, especially for substance use disorder. ASD comorbid with ID alone was not associated with an increased risk of any substance use-related problems (OR 1.1; 95 % CI 0.9–1.3), when all outcomes where regarded as one group.

When the risk was calculated separately for specific outcomes, the risk of substance use disorder was increased (OR 1.8; 95 % CI 1.4–2.2), but the risk of being convicted of a substance-related crime was decreased (OR 0.2; 95 % CI 0.1–0.4). Odds ratios adjusted for parental education, family income and substance use disorder prior to ASD diagnosis showed a similar pattern (Table 2).

In a sensitivity analysis, the risk for substance-related problems was estimated separately for ASD probands who received all neuropsychiatric diagnoses *before* a substance use disorder diagnosis. This suggested that odds ratios were similarly increased in ASD probands with (OR 1.9; 95 % CI 1.6–2.3) and without comorbid ADHD (OR 1.6; 95 % CI 1.4–1.8), while those with comorbid ID appeared to have a decreased risk (OR 0.6; 95 % CI 0.5–0.8). The largest difference in substance use-related problems across comorbidity groups was seen for substance-related crime, which was more likely only among ASD probands with ADHD (OR 1.7; 95 % CI 1.3–2.3). In contrast, ASD probands without ADHD (OR 0.7; 95 % CI 0.5–0.8) were actually less likely to commit substance related crime than were population controls (Table S4).

### Relatives’ Risk of Substance Use-Related Problems

Compared to their matched controls, all relatives of probands had weakly but significantly increased risk for any substance-related problem (Table 3). Full siblings and parents were at weakly to moderately increased risk for all substance use-related problems, including substance-related death. Half-siblings exhibited significantly increased risk for substance-related crime and substance use disorder. The increased risk of substance use-related problems among relatives was present regardless of probands’ comorbidity. Finally, adjustment for socio-demographic covariates did not change results materially (data not shown).

## Discussion

We found that ASD was associated with increased risk for a range of substance use-related problems, and the family data suggested that this was due to shared liability between ASD and substance use-related problems between relatives.

### Autism Spectrum Disorder and Substance Use-Related Problems

Up to now, many clinicians have assumed that substance use-related problems are rare among patients with ASD and, if present, primarily due to comorbid ADHD (Palmqvist et al. 2014). This notion has also been supported by clinical studies. A retrospective chart review of 97 youths with ASD found lower rates of substance use compared to psychiatrically treated controls (3.1 vs. 16.7 %); the three boys with ASD and substance use also had comorbid ADHD (Santosh and Mijovic 2006). Similarly, when 70 adults with ASD were compared to 70 subjects with ADHD, substance use rates were lower among those with ASD than ADHD patients (30 vs. 58 %). (Sizoo et al. 2010) However, both ADHD (Groenman et al. 2013) and psychiatrically treated patients (Mangerud et al. 2014) are at increased risk of substance use-related problems, which makes them less suitable as control subjects. To our knowledge, although prior research argued that substance use-related problems are not an issue among ASD individuals (Ramos et al. 2013), no prior clinical study with ASD patients has compared them to non-ASD population controls. However, more recent twin studies provide a different perspective. Lundström et al. were the first to report that autistic-like traits actually increase the risk of substance abuse (OR 7.4; 95 % CI 3.5–15.7) (Lundstrom et al. 2011), findings which were recently confirmed in Australia (De Alwis et al. 2014). These twin studies focused on autistic-like traits as a behavior pattern within the normal spectrum of social interest and competence and similar to that found in ASD, but without investigating the formal diagnostic criteria for persistence, distress or functional impairment required for a diagnosis. Thus, these studies did not address whether an ASD *diagnosis* was related to substance use-related problems.

Increased risk of substance use-related problems seems to contradict global negative attitudes towards psychoactive substances observed among ASD patients (Ramos et al. 2013). Individuals with ASD may find them helpful to reduce tension and enhance social skills more often than non-ASD controls do (Cludius et al. 2013).

### Cohort Effect and Comorbidity

So, why have the idea that ASD patients are somehow protected from substance use-related problems been quite persistent? One possibility is that substance use-related problems in individuals with ASD were indeed less common in the past, but that some factor(s) caused an increase over time. In fact, substantial time trends in substance use have been described for the general population (Kraus et al. 2015b, a). A cohort effect is one of several factors that may explain such temporal changes in substance use-related problems. The broadening of diagnostic criteria has previously been blamed for increase in ASD prevalence (Lundstrom et al. 2015). Hence, while ASD patients diagnosed after 1996 with ICD-10 appeared to have increased risk of substance use-related problems relative to control subjects, prior more narrow diagnostic practice may have excluded ASD patients with substance use-related issues or assigned other diagnoses to them. Thus, the remaining, narrowly defined ASD patient group will be perceived as being “protected” from substance use-related problems.

The risk of substance use-related problems may still differ across patient subgroups depending on ADHD and ID comorbidity. For example, increased rates of substance use-related problems in ASD have been attributed to comorbid ADHD (Palmqvist et al. 2014). Santosh et al. argued that comorbid ID may protect ASD patients from substance use-related problems (Santosh and Mijovic 2006). This was supported by studies suggesting that substance use-related problems were increased among patients with ASD, but without intellectual disability comorbidities (Hofvander et al. 2009; Sizoo et al. 2010).In this study, the increased risk of substance use-related problems suggested among ASD patients was unlikely to result entirely from comorbid conditions, since probands diagnosed solely with ASD had an almost doubled risk of substance use-related problems compared to non-ASD controls. However, comorbid ADHD and ID seemed to modify the overall risk. For example, co-occurring ADHD was associated with further increased risk of substance use-related problems, whereas ID was associated with a lowered risk. It is possible that the highly increased risk among patients with comorbid ADHD is due to diagnostic bias related to interpretation of ICD by clinicians. Taken literally, ICD-10 does not allow a comorbid ADHD diagnosis in the presence of several other diagnoses; ASD, anxiety-, and mood disorders. Simultaneous diagnoses of ASD and ID is allowed, provided that autistic-like symptoms cannot be explained by ID. Thus, clinicians may be reluctant to assign ASD and ID diagnoses to patients already diagnosed with ASD. In contrast, among patients with ASD and later substance use disorder, clinicians may be more likely to exchange ASD with an ADHD diagnosis. To test this possibility, we performed sensitivity analyses with those ASD patients who assigned with ASD, ADHD and ID before a substance use disorder diagnosis. As a result, it turned out that patients with ASD only and ASD with ADHD are actually on comparable risks of substance use-related problems (OR 1.6 vs. 1.9) and previously described extremely high risk in patents with ASD and ADHD seems to be due diagnostic biases.

### Common Familial Etiology

To further investigate a possible shared familial background to the association between ASD and substance use-related problems, we analyzed the risk among non-ASD relatives of ASD probands. Consistent with prior reports of high rates of alcohol abuse among relatives of ASD patients (Miles et al. 2003) and higher risk of ASD among offspring of parents with alcohol abuse (Sundquist et al. 2014), our results suggested increased risk of substance use-related problems among 1st degree relatives and half-siblings without an ASD diagnosis. This supports a shared familial liability which may in turn reflect one or more possible explanations; shared genetic or environmental factors, or epigenetic mechanisms. First, ASD and substance use-related problems may share genetic risk variants (Zuo et al. 2013). Second, parental substance use disorder may also increase de novo mutation rates, found to be involved in ASD (Sanders et al. 2012). Exposure to psychoactive substances may be related to epigenetic modifications in germ cells (Govorko et al. 2012) and lead to high risk of ASD in offspring. Third, associations between ASD and substance use-related problems may be due to shared environmental factors. For example, exposure to alcohol during pregnancy may lead to fetal alcohol spectrum disorder and autistic-like symptoms within the course of this condition (Stevens et al. 2013). In addition, severely neglected children of parents addicted to psychoactive substance may present symptoms of reactive attachment disorder which, particularly when accompanied with autistic-like symptoms, may increase the probability of receiving an ASD diagnosis (McCullough et al. 2013).

Interestingly, full siblings and parents of ASD probands also had substantially increased risks of substance-related death. This association among parents may be explained by older age at the time of study (median age 47.1 years; IQR 41.4–53.9) enabling sufficient number of outcomes to occur. However, siblings were not older than probands at the time of inclusion (median age 16.9 years; IQR 10.8–22.7). We can only speculate that the same familial factors may be causal in substance use-related problems among ASD probands may lead also to higher risks of substance-related death among their non-ASD relatives. For example, as previously mentioned, a rigid norm-abiding interpersonal style characteristic for ASD may protect from life-threatening activities under the influence of a psychoactive substance.

### Study Strengths and Limitations

Strengths include the large scale population-based design, prospectively collected data from nationwide registries, stratification by comorbid disorders, statistical control for socio-demographic confounders, and analysis of familial aggregation data from relatives. Nevertheless, some limitations deserve comments.

First, information bias should be considered. For example, substance use-related problems may be more likely to be detected among individuals with ASD who do have regular contact with habilitation and mental health services. However, similar results were also obtained from other resources; significantly increased risk for substance-related crime from the National Convictions Register and for alcohol-related somatic disease, diagnosed by other medical specialists. An information bias may also act in an opposite direction. Individuals with ASD had not statistically significant, but slightly higher prevalence of substance use disorder prior to ASD diagnosis then healthy controls.

Second, we could stratify our analysis on comorbid ADHD and ID only when those disorders were diagnosed, but it impossible to rule out that patients with seemingly “pure” ASD have undiagnosed ID, ADHD, or subthreshold ADHD-symptoms involving substance use-related problems. Third, the register-based design needs consideration while comparing absolute estimate rates from our study to those from research using population survey designs. For example, our absolute figures on alcohol use disorder are comparable to 1.5 % rates of alcohol abuse among men and 0.7 % among females estimated in a register-based cohort of Swedish citizens born 1973–1984 (Gauffin et al. 2013). Yet, they evidently differ from the 8.9 % 12-month prevalence of alcohol use disorder found in a Swedish population survey of those aged 15 years and older (WHO 2014). In other words, register-based studies are likely to underestimate the absolute prevalence of substance use disorder since users not in contact with the treatment system are not taken into account (EMCDDA 2004).

Fourth, it was not possible to disentangle if the shared familial background between ASD and substance use-related problems were best explained by genetic or shared environmental factors. Finally, we did not differentiate between Autistic disorder, Asperger´s disorder or Pervasive developmental disorder not otherwise specified which might have yielded different associations. Multi-site studies, although, do question the validity of the demarcation between these three disorders (Lord et al. 2012) and the DSM-5 (APA, 2013), groups them all under the one single category of *autism spectrum disorders*.

## Conclusions

In summary, this large population-based study suggests that individuals with ASD have higher risk of substance use-related problems than population controls; most likely because of a shared familial liability for these conditions. An important implication of our findings concerns diagnostics and treatment strategies in ASD. Increased risk of substance-related problems in ASD suggests attention and preventive measures regarding substance use disorder in this population. Since ASD frequently co-occurs with ADHD and intellectual disability and the trajectories of ASD into substance abuse may differ depending on concomitant conditions, the present results also highlight the need for comprehensive psychiatric examinations upon deciding on treatment strategies for substance use disorder. Our finding of a mediating role of familial risk factors suggest that attempts to prevent or treat substance use disorder in ASD probands should consider also the vulnerabilities of other first degree family members.

## Electronic supplementary material

Below is the link to the electronic supplementary material.

Supplementary material 1 (DOCX 36 KB)
