# Supplementary material for: Increased Risk for Substance Use-Related Problems in Autism Spectrum Disorders: A Population-Based Cohort Study
Source: J Autism Dev Disord. 2016 Oct 12;47(1):80–9. doi: 10.1007/s10803-016-2914-2 (PMC5222913; doi:10.1007/s10803-016-2914-2)
Supplement: Supplementary file 1 — Supplementary material 1 (DOCX 36 KB) [file 10803_2016_2914_MOESM1_ESM.docx]

Table S1: ICD-based definitions of substance use-related problems.

|  | **Medical condition or cause of death** | **Diagnostic code** |
| --- | --- | --- |
| Substance use disorder | Alcohol use disorder | ICD-8 codes 291, 303; ICD-9 codes 291, 303, 305A; ICD-10 code F10 |
|  | Drug use disorder | ICD-8 code 304; ICD-9 code 304; ICD-10 codes F11-F16, F19 |
|  | Tobacco use disorder | ICD-9 code 305B; ICD-10 code F17 |
| Death | Alcohol use disorder | ICD-8 code 303; ICD-9 codes 303, 3050; ICD-10 code F10 |
|  | Alcohol psychosis | ICD-8 code 291; ICD-9 code 291; ICD-10 code F10.5 |
|  | Liver disease and pancreatitis | ICD-8 code 5710; ICD-9 codes 5710-5713; ICD-10 codes K70, K85.2, K86 |
|  | Other alcohol-related diseases | ICD-8 codes; ICD-9 codes 3575, 4255, 5353; ICD-10 codes G62.1, G31.2, G72.1, I42.6, K29.2 |
|  | Alcohol poisoning | ICD-8 codes E860, N980.0; ICD-9 codes E8600, E8601, N90800; ICD-10 codes X45, X65, Y15 with T51.0 |
|  | Drug dependence and poisoning | ICD-9 and ICD-10 EMCDDA definitions ([EMCDDA 2009](#_ENREF_10)), ICD-8 codes 304 except 304.2, E853.0, E856.4, E859.0, E853.9 with N965.0, E853.9 with N969, E850.2 with N965.0, E850.2 with N969, E850.3 with N965.0, E850.3 with N969, E880 with N965.0, E880 with N969 |
| Somatic disease | Disorders explicitly linked to alcohol misuse | ICD-8 codes 57100, 57101; ICD-9 codes 357F, 425F, 535D, 571A-571D; ICD-10 codes G31.2, G62.1, I42.6, K29.2, K70, K85.2, K86.0 |
|  | Liver and pancreatic diseases  with alcohol misuse | ICD-8 code 571, 577; ICD-9 code 571, 577; ICD-10 codes K29, K71-K74  ICD-8 codes 291,303; ICD-9 codes 291, 303, 305A; ICD-10 code F10 |
|  | Gastritis with alcohol misuse | ICD-8 code 535; ICD-9 code 535; ICD-10 codes K70, K85.2, K86  ICD-8 codes 291,303; ICD-9 codes 291, 303, 305A; ICD-10 code F10 |
|  | Polyneuropathy  with alcohol misuse | ICD-9 code 357 ex 357A, 357W; ICD-10 code G62  ICD-8 codes 291,303; ICD-9 codes 291, 303, 305A; ICD-10 code F10 |
|  | Cardiomyopathy  with alcohol misuse | ICD-8 code 425; ICD-9 code 425; ICD-10 code I42  ICD-8 codes 291,303; ICD-9 codes 291, 303, 305A; ICD-10 code F10 |

Table S2: Descriptive data for autism spectrum disorder(ASD) probands, their relatives and matched population controls with their respective relatives.

| **Characteristic** | **Probands** | **Unexposed (control) individuals** | **Full siblings of probands** | **Unexposed full siblings** | **Half-siblings of probands** | **Unexposed half-siblings** | **Parents of probands** | **Unexposed parents** |
| --- | --- | --- | --- | --- | --- | --- | --- | --- |
|  | Patients with ASD  *N=*26,986  *n* (%) | Non-ASD individuals  *N=*1,349,300  *n* (%) | Full siblings of patients with ASD *N=*30,456  *n* (%) | Full siblings of non-ASD individuals *N=*1,306,391  *n* (%) | Half-siblings of patients with ASD *N=*15,946  *n* (%) | Half-siblings of non-ASD individuals *N=*403,214  *n* (%) | Parents of patients with ASD *N=*50,155  *n* (%) | Parents of non-ASD individuals  *N=*1,625,776  *n* (%) |
| Sex |  |  |  |  |  |  |  |  |
| - Male | 19000 (70.4) | 950000 (70.4) | 15700 (51.5) | 677122 (51.8) | 8159 (51.2) | 207477 (51.5) | 24817 (49.5) | 797394 (49.0) |
| - Female | 7986 (29.6) | 399300 (29.6) | 14756 (48.5) | 629269 (48.2) | 7787 (48.8) | 195737 (48.5) | 25338 (50.5) | 828382 (51.0) |
| - Year of birth (year) |  |  |  |  |  |  |  |  |
| - Before 1950 | - | - | - | - | - | - | 5496 (11.0) | 124044 (7.7) |
| - 1950-1959 | - | - | - | - | - | - | 13643 (27.3) | 412159 (25.6) |
| - 1960-1969 | - | - | - | - | - | - | 20873 (41.8) | 721681 (44.8) |
| - 1970-1979 | 1872 (17.4) | 105317 (15.3) | 2225 (14.3) | 84135 (12.5) | 2164 (24.6) | 42532 (19.6) | 9162 (18.4) | 328710 (20.4) |
| - 1980-1989 | 5317 (49.5) | 331638 (48.3) | 7983 (51.4) | 343899 (51.2) | 4518 (51.3) | 116136 (53.6) | 729 (0.0) | 25141 (0.0) |
| - 1990-1999 | 3546 (33.0) | 249337 (36.3) | 5323 (34.3) | 244201 (36.3) | 2117 (24.1) | 58061 (26.8) | - | - |
| - Highest obtained education |  |  |  |  |  |  |  |  |
| - Primary and lower secondary | 6364 (59.3) | 245335 (35.7) | 5984 (38.5) | 236464 (35.2) | 3277 (37.2) | 79044 (36.5) | 7966 (16.0) | 242061 (15.0) |
| - Upper secondary | 3387 (31.6) | 295075 (43.0) | 6325 (40.7) | 288579 (42.9) | 4159 (47.3) | 101941 (47.0) | 24462 (49.0) | 818400 (50.8) |
| - Post-secondary | 638 (5.9) | 70703 (10.3) | 1588 (10.2) | 72414 (10.8) | 684 (7.8) | 18756 (8.7) | 7107 (14.2) | 233849 (14.5) |
| - Postgraduate | 346 (3.2) | 75179 (11.0) | 1634 (10.5) | 74778 (11.1) | 679 (7.7) | 16988 (7.8) | 10285 (20.6) | 315083 (19.5) |
| - Unknown | - | - | - | - | - | - | 83 (0.2) | 2342 (0.1) |
| Maternal age at birth (years) |  |  |  |  |  |  |  |  |
| - <35 | 22416 (83.1) | 1152147 (85.4) | 25466 (83.6) | 1126407 (86.2) | 13471 (84.5) | 339358 (84.2) | 37826 (75.4) | 1218872 (75.0) |
| - ≥35 | 4532 (16.8) | 193285 (14.3) | 4965 (16.3) | 178802 (13.7) | 2458 (15.4) | 63508 (15.8) | 4965 (9.9) | 149814 (9.2) |
| - Unknown | 38 (0.1) | 3868 (0.3) | 25 (0.1) | 1182 (0.1) | 17 (0.1) | 348 (0.1) | 7364 (14.7) | 257090 (15.8) |
| Paternal age at birth (years) |  |  |  |  |  |  |  |  |
| - <35 | 18150 (67.3) | 949481 (70.4) | 20329 (66.7) | 924797 (70.8) | 11205 (70.3) | 282620 (70.1) | 31632 (63.1) | 1035542 (63.7) |
| - ≥35 | 8503 (31.5) | 381562 (28.3) | 10085 (33.1) | 379797 (29.1) | 4724 (29.6) | 120097 (29.8) | 10010 (20.0) | 302841 (18.6) |
| - Unknown | 333 (1.2) | 18257 (1.4) | 42 (0.1) | 1797 (0.1) | 17 (0.1) | 497 (0.1) | 8513 (17.0) | 287393 (17.7) |
| Mother’s region of birth |  |  |  |  |  |  |  |  |
| - Sweden | 22352 (82.8) | 1118931 (82.9) | 25308 (83.1) | 1077605 (82.5) | 13703 (85.9) | 333778 (82.8) | 36716 (73.2) | 1184663 (72.9) |
| - Other Nordic country | 1133 (4.2) | 56288 (4.2) | 1239 (4.1) | 48482 (3.7) | 791 (5.0) | 21192 (5.3) | 3588 (7.2) | 97951 (6.0) |
| - Outside Nordic countries | 3463 (12.8) | 170213 (12.6) | 3884 (12.8) | 179122 (13.7) | 1435 (9.0) | 47896 (11.9) | 2487 (5.0) | 86072 (5.3) |
| - Unknown | 38 (0.1) | 3868 (0.3) | 25 (0.1) | 1182 (0.1) | 17 (0.1) | 348 (0.1) | 7364 (14.7) | 257090 (15.8) |
| Father’s region of birth |  |  |  |  |  |  |  |  |
| - Sweden | 21899 (81.1) | 1098559 (81.4) | 25057 (82.3) | 1061863 (81.3) | 13114 (82.2) | 318086 (78.9) | 36272 (72.3) | 1171286 (72.0) |
| - Other Nordic country | 1018 (3.8) | 47308 (3.5) | 1158 (3.8) | 41132 (3.1) | 727 (4.6) | 17773 (4.4) | 2617 (5.2) | 70419 (4.3) |
| - Outside Nordic countries | 3736 (13.8) | 185176 (13.7) | 4199 (13.8) | 201599 (15.4) | 2088 (13.1) | 66858 (16.6) | 2753 (5.5) | 96678 (5.9) |
| - Unknown | 333 (1.2) | 18257 (1.4) | 42 (0.1) | 1797 (0.1) | 17 (0.1) | 497 (0.1) | 8513 (17.0) | 287393 (17.7) |
| Highest parental education |  | |  | |  | |  | |
| - Primary and lower secondary | 1389 (5.1) | 64103 (4.8) | 1466 (4.8) | 64698 (5.0) | 1250 (7.8) | 27596 (6.8) | 13675 (27.3) | 438550 (27.0) |
| - Upper secondary | 12337 (45.7) | 583601 (43.3) | 13820 (45.4) | 560625 (42.9) | 9385 (58.9) | 222190 (55.1) | 16872 (33.6) | 555754 (34.2) |
| - Post-secondary | 4674 (17.3) | 244966 (18.2) | 5402 (17.7) | 237820 (18.2) | 2198 (13.8) | 64776 (16.1) | 3314 (6.6) | 108876 (6.7) |
| - Postgraduate | 8561 (31.7) | 449181 (33.3) | 9751 (32.0) | 441412 (33.8) | 3112 (19.5) | 88501 (21.9) | 7990 (15.9) | 247168 (15.2) |
| - Unknown | 25 (0.1) | 7449 (0.6) | 17 (0.1) | 1836 (0.1) | 1 (0.0) | 151 (0.0) | 8304 (16.6) | 275428 (16.9) |
| Family income (percentile) |  | |  | |  | |  | |
| - <20 | 6404 (23.7) | 316767 (23.5) | 9931 (32.6) | 410385 (31.4) | 3919 (24.6) | 87734 (21.8) | 3806 (7.6) | 106873 (6.6) |
| - 20-79 | 19283 (71.5) | 939424 (69.6) | 19537 (64.1) | 826776 (63.3) | 11513 (72.2) | 292512 (72.5) | 25050 (49.9) | 859387 (52.9) |
| - >=80 | 1289 (4.8) | 87063 (6.5) | 987 (3.2) | 68063 (5.2) | 512 (3.2) | 22899 (5.7) | 3087 (6.2) | 111700 (6.9) |
| - Unknown | 10 (0.0) | 6046 (0.4) | 1 (0.0) | 1167 (0.1) | 2 (0.0) | 69 (0.0) | 18212 (36.3) | 547816 (33.7) |

Table S3: Odds ratios (with 95% confidence interval) for substance use-related problems in ASD probands**,** stratified by ICD classification at assignment of ASD diagnosis.

| **Substance-related problem** | **Classification cohort** |  |  |  |  |  |
| --- | --- | --- | --- | --- | --- | --- |
|  | **ICD-8/ICD-9** |  |  | **ICD-10** |  |  |
|  | **Patients with ASD**  ***N=*890**  ***n* (%)** | **Non-ASD individuals**  ***N=*44,500 *n* (%)** | **Crude OR (95% CI)** | **Patients with ASD**  ***N=*26,096**  ***n* (%)** | **Non-ASD individuals**  ***N=*1,304,800 *n* (%)** | **Crude OR (95% CI)** |
| Any problem | 17 (1.9) | 2259 (5.0) | 0.4 (0.2-0.6)*** | 1062 (4.1) | 15384 (1.2) | 3.8 (3.6-4.1)*** |
| - Substance use disorder | 13 (1.5) | 1213 (2.7) | 0.4 (0.2-0.6)* | 967 (3.7) | 9015 (0.7) | 5.9 (5.5-6.3)*** |
| Alcohol | 10 (1.1) | 930 (2.0) | 0.5 (0.3-0.9)* | 564 (2.1) | 6589 (0.5) | 4.5 (4.1-4.9)*** |
| Drugs | 3 (0.3) | 392 (0.8) | 0.5 (0.3-1.0) | 576 (2.2) | 3246 (0.2) | 9.5 (8.7-10.4)*** |
| Tobacco | 1 (0.1) | 12 (0.0) | 0.4 (0.1-1.2) | 16 (0.1) | 122 (0.0) | 6.6 (3.9-11.1)*** |
| - Crime | 6 (0.7) | 1365 (3.0) | 0.2 (0.1-0.5)*** | 253 (1.0) | 8322 (0.6) | 1.5 (1.4-1.8)*** |
| - Somatic disease | 7 (0.0) | 59 (0.0) | -† | 7 (0.0) | 46 (0.0) | 7.6 (3.4-16.9)*** |
| - Death | 0 (0.0) | 13 (0.0) | -† | 6 (0.0) | 78 (0.0) | 3.9 (1.7-8.8)** |

* *p* value <0.05;** *p* value <0.01; *** *p* value <0.001; †- OR and 95% CI were not calculable due to no observations.

Table S4. Odds ratios (with 95% confidence interval) for substance use-related problems in ASD probands compared to matched non-ASD controls among individuals with neuropsychiatric disorders diagnosed prior to substance use disorder included. Results stratified by comorbidity

| **Substance-related problem** | **Bivariate analysis** | | | | **Multivariate analysis #** | | | |
| --- | --- | --- | --- | --- | --- | --- | --- | --- |
|  | **Comorbidity** | | | | **Comorbidity** | | | |
|  | **None** | **ADHD** | **ID** | **ADHD + ID** | **None** | **ADHD** | **ID** | **ADHD + ID** |
|  | **Crude OR (95% CI)** | **Crude OR (95% CI)** | **Crude OR (95% CI)** | **Crude OR (95% CI)** | **Adjusted OR (95% CI)** | **Adjusted OR (95% CI)** | **Adjusted OR (95% CI)** | **Adjusted OR (95% CI)** |
| Any problem | 1.6 (1.4-1.8)*** | 1.9 (1.6-2.3)*** | 0.6 (0.5-0.8)*** | 1.0 (0.7-1.5) | 1.9 (1.6-2.1)*** | 1.9 (1.6-2.3)*** | 0.7 (0.5-0.9) | 1.0 (0.7-1.5) |
| - Substance use disorder | 2.4 (2.1-2.8)*** | 2.2 (1.7-2.8)*** | 1.0 (0.8-1.3) | 1.4 (0.9-2.3) | 3.1 (2.7-3.6)*** | 2.5 (2.0-3.2)*** | 1.2 (0.9-1.6) | 1.6 (1.0-2.6)* |
| - - Alcohol | 2.1 (1.8-2.5)*** | 1.6 (1.2-2.3)** | 0.8 (0.6-1.2) | 1.1 (0.6-2.1) | 2.6 (2.2-3.1)*** | 1.9 (1.4-2.6)*** | 1.0 (0.7-1.5) | 1.2 (0.6-2.3) |
| - - Drugs | 3.3 (2.7-4.0)*** | 3.5 (2.5-4.9)*** | 1.3 (0.8-1.9) | 2.1 (1.1-4.0)*** | 4.6 (3.8-5.7)*** | 4.3 (3.1-6.0)*** | 1.6 (1.1-2.5)* | 2.6 (1.3-4.8)** |
| - - Tobacco | 1.8 (0.4-7.2) | 2.1 (0.3-15.4) | 6.0 (1.8-19.9)* | - † | 2.1 (0.5-8.6) | 2.3 (0.3-17.0) | - † | - † |
| - Crime | 0.7 (0.5-0.8)** | 1.7 (1.3-2.2)*** | 0.2 (0.1-0.3)*** | 0.7 (0.4-1.4) | 0.7 (0.6-0.9)* | 1.6 (1.2-2.1)** | 0.2 (0.1-0.3)*** | 0.7 (0.4-1.3) |
| - Death | 3.4 (1.0-10.8)* | -† | -† | -† | - † | - † | - † | -† |
| - Somatic disease | 1.8 (0.2-13.1) | - † | 11.1 (2.4-51.4)** | -† | 2.6 (0.4-19.5) | - † | - † | -† |

ADHD, attention deficit hyperactivity disorder; ID, intellectual disability; * *p* value <0.05;** *p* value <0.01; *** *p* value <0.001; †- OR and 95% CI were not calculable due to no observations;
